# Understanding Vascular Anatomy is Key to Successful Endovascular Treatment of Pancreaticoduodenal Artery Aneurysms

**DOI:** 10.3400/avd.cr.20-00011

**Published:** 2020-09-25

**Authors:** Koji Hirano, Toshiya Tokui, Bun Nakamura, Ryosai Inoue, Reina Hirano, Yasumi Maze, Shuji Chino, Hisato Ito, Yu Shomura, Motoshi Takao

**Affiliations:** 1 Department of Thoracic and Cardiovascular Surgery, Ise Red Cross Hospital; 2 Department of Radiology, Ise Red Cross Hospital; 3 Department of Thoracic and Cardiovascular Surgery, Mie University Hospital

**Keywords:** pancreaticoduodenal artery aneurysm, endovascular treatment, celiac trunk obstruction

## Abstract

Pancreaticoduodenal artery aneurysm (PDAA) is a rare disease without treatment guidelines. We present two patients with PDAA. The first patient was a 70-year-old man with a pseudoaneurysm in the anterior superior pancreaticoduodenal artery (ASPDA), for which we achieved exclusion by endovascular coil embolization. The second patient was a 63-year-old woman with a PDAA in the ASPDA with celiac axis obstruction. Endovascular coil embolization of the aneurysm and the ASPDA was successful without visceral organ ischemia. Endovascular treatment is effective for PDAAs, but careful evaluation of collateral circulation is vital in PDAAs with celiac axis obstruction.

## Introduction

Pancreaticoduodenal artery aneurysm (PDAA) is a rare pathology accounting for 1.5%–2% of visceral artery aneurysms^1,2)^; however, 20%–45% of PDAAs are ruptured at presentation, and the size of the ruptured PDAA is often smaller than 2 cm.^3,4)^ These findings indicate that PDAA is more prone to rupture than other visceral artery aneurysms. Therefore, early diagnosis and planning of the treatment strategy is necessary, regardless of the aneurysm’s size. In this report, we present two patients with this potentially fatal disease who were successfully treated with endovascular techniques, and we provide a short review discussing the key factors to consider when performing endovascular treatment of PDAA.

## Case Report

### Patient 1

**Figure 1:**
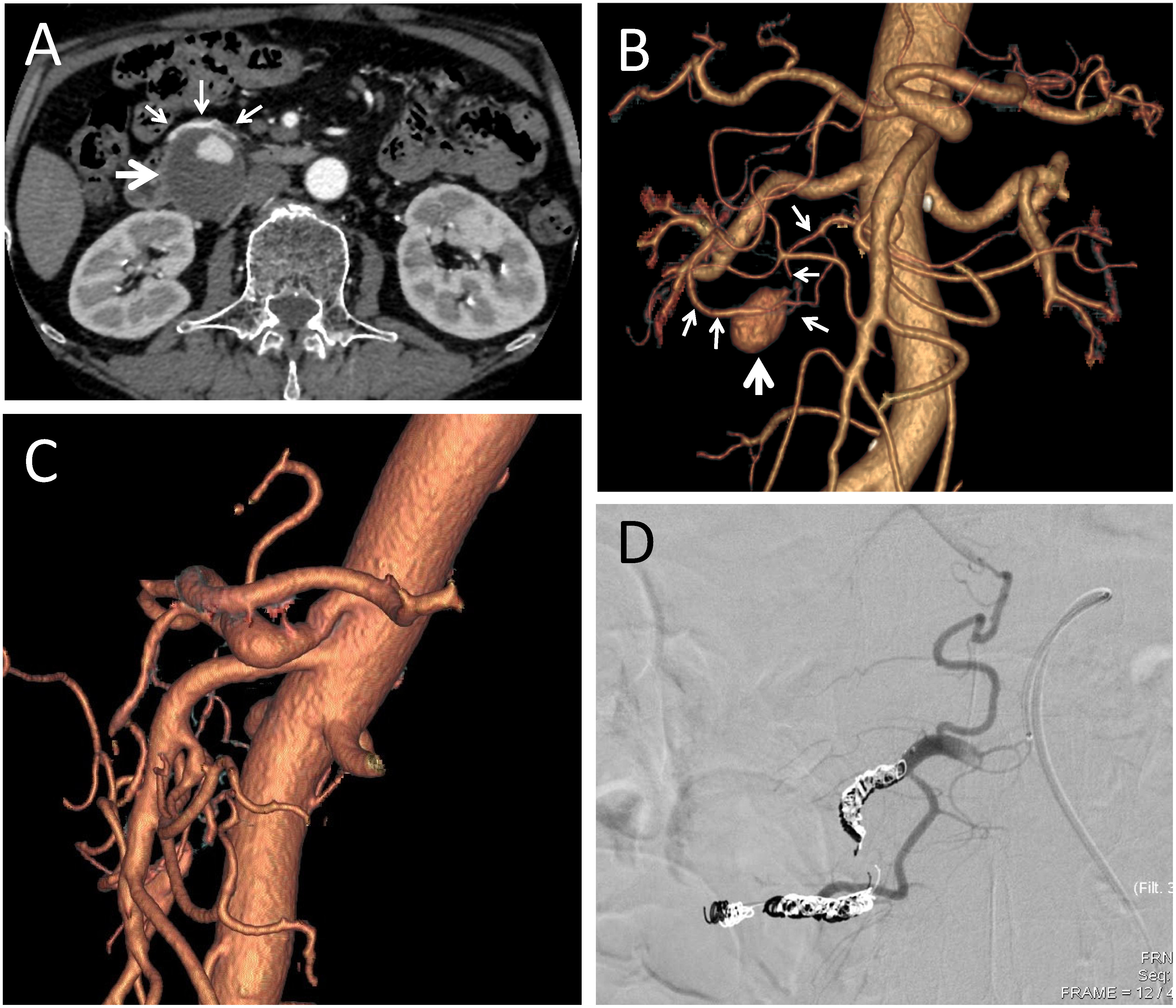
Fig. 1 Images for patient 1. (**A**) Preoperative computed tomography (CT) slice demonstrating the pancreaticoduodenal artery aneurysm (large arrow). (**B**) Three-dimensional (3D)-CT image of the aneurysm (large arrow) and the adjacent vascular anatomy. (**C**) Lateral 3D-CT image demonstrating no obstructive lesion at the celiac artery axis. (**D**) Angiographic image after coil embolization of the anterior superior pancreaticoduodenal artery (ASPDA). The aneurysm is no longer visible. Small arrows: ASPDA.

A 70-year-old man with laryngeal cancer underwent contrast-enhanced computed tomography (CT) to identify metastasis, and a pseudoaneurysm with a diameter of 40 mm was found incidentally at the anterior superior pancreaticoduodenal artery (ASPDA) (**Figs. 1A** and **1B**). Liver transaminase and pancreatic enzyme levels in his blood laboratory testing were normal. Because CT showed no celiac axis stenosis and occlusion (**Fig. 1C**), and, therefore, the ASPDA was not a collateral artery feeding any visceral organs, we selected endovascular coil embolization of the ASPDA to treat the aneurysm. The following is the detailed sequence of the procedure: First, under local anesthesia, superior mesenteric artery (SMA) arteriography was performed with a 4.2-Fr catheter (Excellent EN; Hanako Medical Co., Ltd., Saitama, Japan) inserted from the right femoral artery to confirm the location of the aneurysm and the surrounding vascular anatomy. Next, a 2.0-Fr microcatheter with two radio-opaque markers (Excelsior™ 1018; Stryker Corporation, Kalamazoo, MI, USA) was advanced with a guide wire into the ASPDA, passing over the aneurysm and placed with its tip located distal to the aneurysm. Then, coil embolization was performed using six coils with a diameter of 6 mm (Tornado; Cook Medical, Bloomington, IN, USA). Next, we retracted the microcatheter to a point proximal to the aneurysm and continued the coil embolization using five coils with diameters of 5–6 mm (Tornado; Cook Medical). Finally, successful exclusion of the aneurysm was confirmed by completion angiography (**Fig. 1D**). The patient was uneventfully discharged the next day, and no recurrence of the aneurysm was seen on follow-up CT scans.

### Patient 2

**Figure 2:**
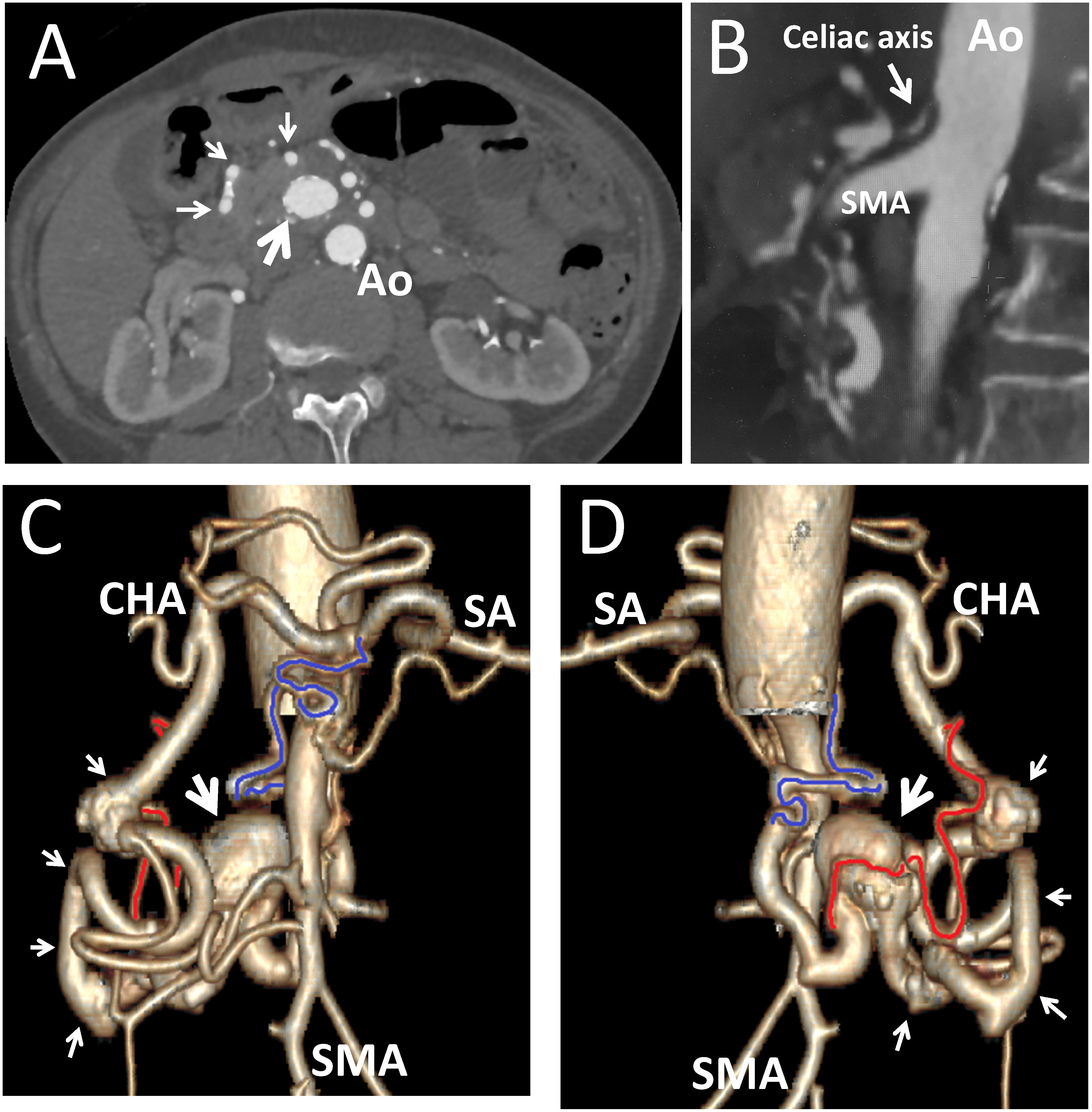
Fig. 2 Preoperative computed tomography (CT) images for patient 2. (**A**) Preoperative CT slice demonstrating the pancreaticoduodenal artery aneurysm (large arrow). The dilated anterior superior pancreaticoduodenal artery (ASPDA) is also visible (small arrows). (**B**) The axis of the celiac artery was occluded. (**C**, **D**) View from the front (**C**) and the back (**D**) in the preoperative three-dimensional CT images. In addition to the ASPDA (small arrows), the posterior superior pancreaticoduodenal artery (red lines) and the dorsal pancreatic artery (blue lines) are visible as collateral pathways connecting the SMA and the celiac artery. Large arrow: the aneurysm.

**Figure 3:**
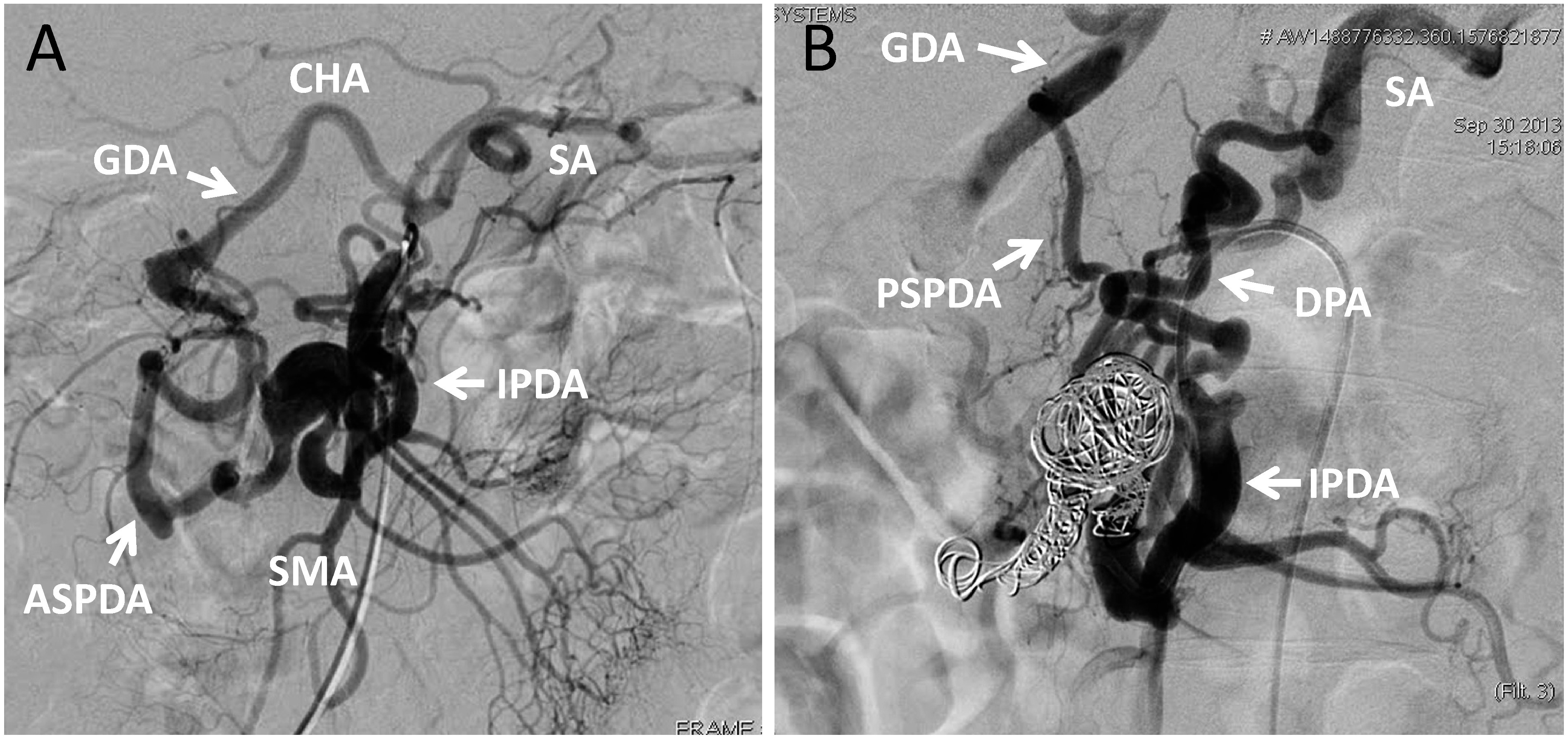
Fig. 3 Coil embolization for patient 2. (**A**) Superior mesenteric arteriography before the procedure. (**B**) Post-procedural completion angiography. The PSPDA and the DPA provide collateral pathways for blood flow to the CHA and the SA, respectively.

CHA: common hepatic artery; SA: splenic artery; SMA: superior mesenteric artery; GDA: gastroduodenal artery; ASPDA: anterior superior pancreaticoduodenal artery; PSPDA: posterior superior pancreaticoduodenal artery; IPDA: inferior pancreaticoduodenal artery; DPA: dorsal pancreatic artery

A 63-year-old woman with chronic rheumatoid arthritis and dyslipidemia underwent abdominal ultrasonography for a medical check-up, which incidentally revealed a mass shadow near the pancreas. Subsequent enhanced CT detected a fusiform-type aneurysm with a diameter of 19 mm at the ASPDA. The celiac axis appeared to be occluded, likely owing to compression by the median arcuate ligament, and distal circulation of the celiac artery appeared to be provided by the SMA through the ASPDA because tortuous and dilated transformations in the ASPDA were also confirmed (**Figs. 2A–2D**). Even in this case, we considered endovascular treatment possible because detailed evaluation of the CT images revealed two other collateral pathways connecting the SMA and branches of the celiac artery, which referred to the posterior superior pancreaticoduodenal artery (PSPDA) and the dorsal pancreatic artery (**Figs. 2C** and **2D**). In other words, these collaterals could provide blood flow to the celiac artery after embolizing the aneurysm and the ASPDA. The following is the detailed sequence of the procedure: First, SMA arteriography was performed with a 4-Fr catheter (CNS1; Terumo Clinical Supply, Gifu, Japan) inserted from the right femoral artery to identify the location of the aneurysm and the surrounding vascular anatomy (**Fig. 3A**). Next, preceded by a guide wire, we advanced a 2.0-Fr microcatheter with two radio-opaque markers (Excelsior™ 1018; Stryker Corp.) into the ASPDA through the 4-Fr catheter, passing through the aneurysm and placed with its tip distal to the aneurysm. Then, coil embolization was performed using the following coils: one detachable coil with a diameter of 8 mm and length of 20 cm (Target XL™; Stryker Corp.) and four coils with diameters of 8–10 mm (Tornado; Cook Medical). Then, we retracted the microcatheter back to the aneurysm and performed coil embolization of the aneurysm itself using three detachable coils with diameters of 18–24 mm and lengths of 30–40 cm (Target XL™; Stryker Corp.). Next, we retracted the microcatheter further back, to the ASPDA proximal to the aneurysm, and continued the coil embolization using three coils with diameters of 5–6 mm (Tornado; Cook Medical), being careful to not occlude the PSPDA, which originated from proximal to the aneurysm. The final angiography showed that the contrast agent pooled slightly within the aneurysm (**Fig. 3B**). However, we did not add more coils because of the risk of occluding the PSPDA. Subsequently, post-treatment enhanced CT showed occlusion of the aneurysm and the ASPDA and patency of the PSPDA, the dorsal pancreatic artery, and the celiac artery branches. The patient was discharged the next day without signs associated with visceral organ ischemia. No recurrence of the aneurysm was observed in follow-up CT scans.

Ao: aorta; CHA: common hepatic artery; SA: splenic artery; SMA: superior mesenteric artery

## Discussion

PDAAs exhibit greatly varying etiologies, such as atherosclerotic degeneration, post-pancreatitis pseudoaneurysm, inflammatory arteriopathy, trauma, infection, connective tissue disorders, and segmental arterial mediolysis.^5,6)^ Another notable factor related to developing a PDAA is increased blood flow in the pancreatic arcade, which comprises the gastroduodenal artery, ASPDA, PSPDA, and the inferior pancreaticoduodenal artery, when the celiac artery or the SMA is stenosed or occluded because of compression by the median arcuate ligament, atherosclerotic plaques, or aortic or local dissections.^1,5)^ This scenario may be the most likely cause for developing PDAA and reportedly causes from 46% to 67% of all PDAAs.^4,7)^ In our patient 1, the PDAA presented with no apparent etiology. The possibility exists that the patient presented with an unknown history of trauma, infection, or pancreatitis because the aneurysmal structure was a pseudoaneurysm. In patient 2, according to preoperative CT findings, the PDAA should have been related to the celiac axis obstruction caused by the median arcuate ligament.

PDAAs sometimes cause abdominal pain, abdominal discomfort, or symptoms associated with pancreatitis^2)^ or, in extremely rare cases, symptoms accompanied by duodenal compression caused by the markedly expanded aneurysm.^8)^ However, in most cases, including in our patients, PDAAs show no symptoms and are detected incidentally during imaging examinations performed for other purposes.^1)^

The factors predicting PDAA rupture have not been clarified; therefore, no treatment guidelines, particularly regarding the timing of intervention, are currently available. However, according to previous studies, PDAAs tend to rupture more easily than other visceral arterial aneurysms, at a rate of 20%–45% of PDAAs, and the size is often smaller than 2 cm at the time of rupture.^3,4)^ Therefore, some authors recommend that PDAAs be treated aggressively when identified, regardless of their size.

During the last decade, endovascular repair became the first-line treatment for PDAAs.^1,4)^ However, not all PDAAs can be repaired with endovascular techniques alone. Successful endovascular repair for PDAA requires the correct diagnosis, including the size, shape, location, and accessibility of the PDAA, which can be obtained by preoperative CT. However, preoperative CT is not always possible because of unstable hemodynamics in some patients with ruptured aneurysms. Even if CT is possible, an expanded hematoma could change the surrounding anatomy—for example by collapse of the adjacent arteries, which might be used to access the ruptured aneurysm.^7)^ Additionally, minimally stable patient blood pressure is needed to complete an entire endovascular procedure in patients with ruptured PDAAs. Nevertheless, we believe that these cases are relatively rare, even for patients with ruptured PDAAs, and that most patients can be managed using endovascular techniques. Another crucial factor when choosing endovascular treatment for PDAAs is whether the PDAA is associated with celiac axis obstruction, caused mostly by compression from the median arcuate ligament. If no obstruction is present in the celiac axis, as was seen in our patient 1, coil embolization to the parent arteries from which the PDAA developed results in only a minor effect on circulation to the visceral organs. Therefore, successful results are expected with a relatively simple endovascular technique such as coil embolization to the parent arteries. Conversely, when the celiac axis is stenosed or occluded, the pancreatic arcade is an alternative collateral pathway for feeding the adjunctive visceral organs of the celiac artery such as the liver, gall bladder, and spleen. In this case, evaluating whether the parent arteries of the PDAA can safely be occluded without causing visceral organ ischemia is essential. Theoretically, if such a risk exists, additional surgical or endovascular treatments, namely bypass formation to the common hepatic artery, angioplasty of the stenotic celiac axis, or sectioning of the median arcuate ligament, should be considered concomitantly with coil embolization.^1,9)^ Alternatively, as previously reported, measuring blood pressure in the hepatic artery when treating PDAAs with celiac axis occlusion and evaluating whether sufficient blood flow is maintained after coil embolization of the parent arteries may be a reasonable method to safely decide whether to add revascularization of the celiac artery.^10)^ In contrast, some reports mention that interventions to address celiac axis lesions are unnecessary and that PDAAs can be managed with coil embolization alone without causing visceral organ ischemia.^4,7)^ These reports suggest that other vascular networks are present, such as the esophageal artery–left gastric artery–common hepatic artery pathway or the superior mesenteric artery–dorsal pancreatic artery–splenic artery–common hepatic artery pathway, that act as collateral vessels after coil embolization of pancreaticoduodenal arteries.^4)^ In accordance with these perspectives, the key factor for successful endovascular repair of PDAAs without visceral organ ischemia is predicting whether sufficient collateral blood flow will be maintained after the treatment. Accordingly, evaluating the vascular anatomy around the PDAA with preoperative three-dimensional-CT is essential, and during endovascular treatment, a balloon occlusion test with pressure monitoring in the common hepatic artery or with selective angiography in the SMA may be helpful to assess collateral circulation. Additionally, the final decision regarding whether to treat the celiac lesion should be made according to the results of post-embolization angiography (i.e., whether branches of the celiac artery remain visible.). In our patient 2, we found two collateral vessels other than the ASPDA (the PSPDA and the dorsal pancreatic artery) using detailed evaluation of preoperative CT images, and we were careful to not occlude these two collateral vessels when embolizing the aneurysm and the ASPDA. Furthermore, we finally confirmed preserved circulation in branches of the celiac artery by the final angiography. Consequently, this patient did not develop visceral organ ischemia. Such careful management is believed to lead to successful endovascular treatment of PDAAs with celiac axis obstruction.

Another concern when a celiac lesion is left untreated at the time of PDAA repair is aneurysmal recurrence secondary to residual increased blood flow in the pancreatic arcade. To the best of our knowledge and according to previous reports, recurrence is unlikely to occur, as in our patient 2, even if the celiac lesion remains untreated.^4)^ However, this finding is based on limited experience and few reports, and caution is warranted; continuous follow-up is mandatory.

## Conclusion

We managed two patients with a relatively rare vascular disease, PDAA, who were treated with endovascular intervention. For successful endovascular treatment, precisely evaluating the collateral circulation when the PDAA arises in association with celiac axis obstruction is necessary.
